# Does Drain Position and Duration Influence Outcomes in Patients Undergoing Burr-Hole Evacuation of Chronic Subdural Hematoma? Lessons from a UK Multicenter Prospective Cohort Study

**DOI:** 10.1093/neuros/nyy366

**Published:** 2018-08-29

**Authors:** Laurence Johann Glancz, Michael Tin Chung Poon, Ian Craig Coulter, Peter John Hutchinson, Angelos Georgiou Kolias, Paul Martin Brennan

**Affiliations:** 1 Department of Neurosurgery, Queen's Medical Centre, Derby Road, Nottingham, NG7 2UH, UK; 2 Translational Neurosurgery, Centre for Clinical Brain Sciences, University of Edinburgh, Edinburgh, United Kingdom; 3 Department of Neurosurgery, Royal Victoria Infirmary, Newcastle, United Kingdom; 4 Division of Neurosurgery, Department of Clinical Neurosciences, University of Cambridge, Addenbrooke's Hospital, Cambridge, United Kingdom; 5 Surgery Theme, Cambridge Clinical Trials Unit, Cambridge Biomedical Campus, Cambridge, United Kingdom

**Keywords:** Burr-hole craniostomy, Chronic subdural hematoma, Drains, Outcome, Recurrence

## Abstract

**Background:**

Drain insertion following chronic subdural hematoma (CSDH) evacuation improves patient outcomes.

**Objective:**

To examine whether this is influenced by variation in drain location, positioning or duration of placement.

**Methods:**

We performed a subgroup analysis of a previously reported multicenter, prospective cohort study of CSDH patients performed between May 2013 and January 2014. Data were analyzed relating drain location (subdural or subgaleal), position (through a frontal or parietal burr hole), and duration of insertion, to outcomes in patients aged >16 yr undergoing burr-hole drainage of primary CSDH. Primary outcomes comprised modified Rankin scale (mRS) at discharge and symptomatic recurrence requiring redrainage within 60 d.

**Results:**

A total of 577 patients were analyzed. The recurrence rate of 6.7% (12/160) in the frontal subdural drain group was comparable to 8.8% (30/343) in the parietal subdural drain group. Only 44/577 (7.6%) patients underwent subgaleal drain insertion. Recurrence rates were comparable between subdural (7.7%; 41/533) and subgaleal (9.1%; 4/44) groups (*P* = .95). We found no significant differences in discharge mRS between these groups. Recurrence rates were comparable between patients with postoperative drainage for 1 or 2 d, 6.4% and 8.4%, respectively (*P* = .44). There was no significant difference in mRS scores between these 2 groups (*P* = .56).

**CONCLUSION:**

Drain insertion after CSDH drainage is important, but position (subgaleal or subdural) and duration did not appear to influence recurrence rate or clinical outcomes. Similarly, drain location did not influence recurrence rate nor outcomes where both parietal and frontal burr holes were made. Further prospective cohort studies or randomized controlled trials could provide further clarification.

**ABBREVIATIONS**
BHC: burr-hole craniostomy
CSDH: chronic subdural hematoma
mRS: modified Rankin scale
TDC: twist-drill craniostomy

The benefit of insertion of a closed subdural drainage system at the time of burr-hole drainage of symptomatic chronic subdural hematoma (CSDH) has been demonstrated in a randomized controlled trial^1^ and 2 meta-analyses.^2,3^ A multicenter audit of CSDH treatment in the UK and Ireland confirmed the benefit of drain insertion in terms of symptomatic CSDH recurrence requiring reoperation within 60 d of the index surgery.^4^ However, that same study demonstrated significant variation in the details of surgical management of symptomatic CSDH between surgeons.

There is a paucity of literature examining the precise details of drain insertion and whether location, position, and duration of postoperative drainage affect outcomes. The aim of this study was therefore to determine the effect of these drain variables on clinical outcomes by performing a subgroup analysis of patients in the national audit.

## METHODS

### Participants and Study Settings

The study methodology has been described in detail previously.^4,5^ Briefly, a multicenter, prospective cohort study was conducted to determine differences in operative and perioperative strategies for managing patients with CSDH, as well as clinical outcomes. “Study participants were enrolled at 26 of the 33 UK and Ireland Neurosurgical Units (NSUs) between May 2013 and January 2014. Eligibility criteria were age >16 yr, presentation with a primary or recurrent CSDH confirmed on cranial imaging, and referral to a participating NSU”.^4^ Patient demographics, baseline characteristics including medical conditions and relevant medications, alongside details of pre-, intra-, and postoperative management were recorded by the local clinical teams. Symptomatic recurrence was defined as repeat surgical drainage within 60 d of index admission. “The decision to proceed with revision surgery was at the discretion of the patient's consultant neurosurgeon, based on clinical symptoms, correlated with imaging”.^4^ The modified Rankin scale (mRS) score and destination at discharge from the NSU, morbidity and mortality, and duration of stay in the NSU were also recorded. The following mRS was used: 0–no symptoms; 1–no significant disability (able to carry out all usual activities, despite some symptoms); 2–slight disability (able to look after own affairs without assistance, but unable to carry out all previous activities); 3–moderate disability (requires some help, but able to walk unassisted); 4–moderately severe disability (unable to attend to own bodily needs without assistance, and unable to walk unassisted); 5–severe disability (requires constant nursing care and attention, bedridden, incontinent); 6–dead.

The study protocol was approved nationally by the Academic Committee of the Society of British Neurological Surgeons. Individual participating NSUs were responsible for controlling their own contributory data, and also had local governance approvals in place. Individual patient consent was not required and therefore not sought for this study.

Patients from the cohort study who were transferred to an NSU and underwent primary burr-hole craniostomy (BHC) with drain placement for either unilateral or bilateral CSDH were included in this subgroup analysis. Patients who had undergone previous drainage of an ipsilateral CSDH were excluded. Patients who underwent simultaneous drainage of bilateral CSDH were included in our analyses, as previous analyses of this dataset demonstrated no association between unilateral/bilateral CSDH and the same outcomes of interest.^4^ Recurrence occurring on one side in a patient who underwent drainage of bilateral CSDH was considered as a single recurrence.

For those patients undergoing drain placement at the time of surgery, researchers recorded whether this had been placed in the subdural or subgaleal space (drain location). Drain position (insertion via frontal or parietal burr hole), and duration of postoperative drainage in days were recorded. Of the entire cohort, 33 patients had a single burr-hole operation with drain insertion; these patients were excluded from the drain-position analysis. However, single-burr-hole operations were included in the drain location and duration analyses, when relevant complete data were available.

### Statistical Analysis

Chi-squared, Fisher's exact, parametric and non-parametric tests were used, when appropriate, to compare baseline clinical characteristics. These comparisons were made for each subgroup analysis (drain location, drain position, and duration of drain). We used the Mantel–Haenszel method to examine potential confounders to the association of interest. Multiple logistic regression models were used to calculate adjusted odds ratios. Variables entered into the final multivariate analyses were those that were shown to influence the association of interest in univariate analyses, or putative factors for the outcome of interest. Putative factors entered into the multivariate analyses for recurrence were age, gender, preoperative GCS, preoperative antiplatelet medication use, and preoperative warfarin use. In addition to these factors, dichotomized admission mRS and bed rest were entered into multivariate analyses for functional outcome. Functional outcome was the discharge mRS categorized into favorable (mRS 0-3) and unfavorable (mRS 4-6) outcomes. Likelihood ratio tests were used to determine the model of choice. Patients with missing data were excluded if the missing data were relevant to that particular analysis. Stata version 13.0 (StataCorp LP, College Station, Texas) was used for all analyses.

## RESULTS

**FIGURE 1.**
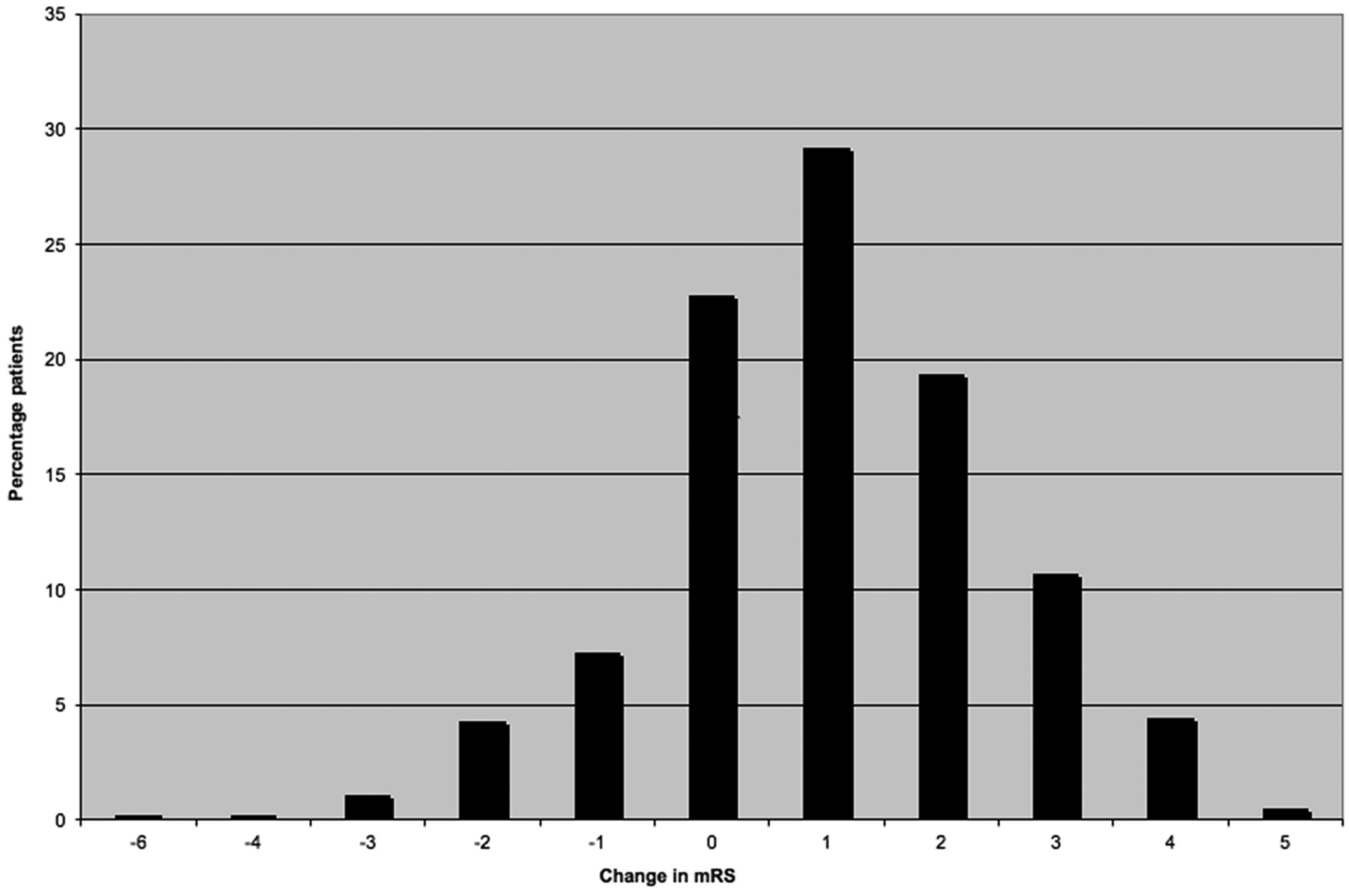
Change in mRS after burrhole craniostomy and drain placement.

Data from 1205 patients were collected in the national audit. Of these, 577 patients had burr-hole drainage of their CSDH with drain insertion and were identified for inclusion in our subgroup analysis; details of their baseline, perioperative, and operative/postoperative characteristics are detailed in Table 1. When comparing admission with postoperative mRS, 64.2% patients showed improvement, 22.8% were the same, and 13% worsened. A detailed breakdown of the changes in mRS is detailed in Figure 1. Overall, the rate of symptomatic CSDH recurrence requiring reoperation within 60 d was 7.8%.

**TABLE 1.** Descriptive Baseline, Perioperative, Operative, and Postoperative Characteristics of 577 Patients Undergoing Burr-Hole Drainage of CSDH With Drain Insertion.

Baseline characteristics	n (%)	Perioperative clinical characteristics	n (%)	Operative characteristics	n(%)
Total number of patients	577	Preoperative platelet transfusion	56 (9.7)	Operative laterality	
Median age in years (IQR)	78 (98-85)	Preoperative vitamin K	91 (15.8)	Left	221 (38.3)
Sex		Preoperative Fresh Frozen Plasma	13 (2.3)	Right	204 (35.4)
Female	183 (31.7)	Preoperative GCS (median, IQR)	14 (13-15)	Bilateral	138 (23.9)
Male	394 (68.3)	GCS 3-8	24 (4.2)	Unknown/Missing	14 (2.4)
Comorbidities		GCS 9-12	78 (13.5)	Anesthesia	
Diabetes mellitus	99 (17.2)	GCS 13-15	475 (82.3)	Local	42 (7.3)
Dementia	64 (11.1)	CSDH laterality		General	535 (92.7)
Chronic Obstructive Pulmonary Disease	35 (6.1)	Left	201 (34.8)	Drain location	
Cerebrovascular event	95 (16.5)	Right	185 (32.1)	Subdural	533 (92.4)
Ischemic heart disease	147 (25.5)	Bilateral	177 (30.7)	Subgaleal	44 (7.6)
Arrhythmia	117 (20.3)	Unknown/missing	14 (2.4)	Subdural drain placement	
Epilepsy	23 (4.0)	CSDH density on pre-operative CT scan		Frontal	176 (33.0)
CSF shunt	6 (1.0)	Hypodense	165 (28.6)	Posterior	315 (59.1)
Malignancy	49 (8.5)	Isodense	120 (20.8)	Unknown	42 (7.9)
Metallic heart valve	11 (1.9)	Mixed	292 (50.6)	Duration of drain placement	
History of head injury within 3 mo	361 (62.6)			1 d	157 (27.2)
Preoperative antithrombotics	245 (42.5)			2 d	392 (67.9)
Antiplatelet medication	137 (23.7)			3 + d	28 (4.9)
Warfarin	108 (18.7)			Bed rest	
Others	9 (1.6)			No specific instructions	215 (37.3)
Admission mRS				1-12 h	37 (6.4)
mRS 0-3	333 (57.7)			12-24 h	231 (40.0)
mRS 4-5	244 (42.3)			24-48 h	85 (14.7)
				48 + h	9 (1.6)

### Drain Location and Position

The majority of patients in our analysis underwent subdural drain insertion (533/577; 92%). The recurrence rate of 7.7% (41/533) in the subdural drain group was not significantly different from 9.1% (4/44) in the subgaleal group (*P* = .95, Table 2). Placement of the subdural drain via either a frontal or parietal burr hole (when 2 burr-holes were performed) resulted in comparable recurrence rates, 6.7% and 8.8%, respectively (*P* = .48, Table 2). There were no significant differences in discharge mRS between the subdural and subgaleal, or between the frontal and parietal subdural drain groups (Table 2).

**TABLE 2.** Outcome Summary Table According to Drain Location, Position, and Duration

	Location (n = 577)			Subdural position (n = 527)			Duration (n = 549)		
	Subdural drain (n = 533)	Subgaleal drain (n = 44)	*P* value	Frontal (n = 179)	Parietal (n = 343)	*P* value	1 d (n = 157)	2 d (n = 392)	*P* value
Recurrence			.95			.48			.44
No	479 (80.9)	39 (88.6)		160 (89.4)	307 (89.5)		142 (90.5)	352 (89.8)	
Yes	41 (7.7)	4 (9.1)		12 (6.7)	30 (8.7)		10 (6.4)	33 (8.4)	
Unknown/missing	13 (2.4)	1 (2.3)		7 (3.9)	6 (1.7)		5 (3.2)	7 (1.8)	
Discharge mRS			.97			.60			.56
mRS 0-3	405 (76.0)	33 (75.0)		139 (77.7)	263 (76.7)		122 (77.7)	297 (75.8)	
mRS 4-6	114 (21.4)	10 (22.7)		33 (18.4)	73 (21.3)		30 (19.1)	87 (22.2)	
Unknown/missing	14 (2.6)	1 (2.3)		7 (3.9)	7 (2.0)		5 (3.2)	8 (2.0)	

Multivariate analyses revealed that when adjusting for the pre-specified variables that affect recurrence rate and functional outcome, there appeared to be no correlation between different drain location/position and outcomes (Tables 3 and 4). A comparison of complications and mortality for drain location and position is illustrated in Table 5. In the univariate analyses, there were no significant differences between the 2 groups except for new neurological deficit that was seen more commonly when the drain was placed via a frontal rather than parietal burr hole (4.9% vs 1.7%, respectively, *P* = .04). Complications according to the duration of drain are shown in Table 6. Self-reported complications included 1 intracerebral hematoma (0.2%), 3 acute subdural hematomas (0.6%), and 2 subdural empyemas (0.4%) in the subdural drain group. None of these complications were reported in the subgaleal drain group.

**TABLE 3.** Multiple Logistic Regression Analysis With Adjusted Odds Ratios for Symptomatic Recurrence Requiring Re-operation Within 60 d According to Drain Location, Position, and Duration in Patients With Complete Data for Analysis.

Location of drain (n = 563)				Position of drain (n = 509)				Drain duration (n = 537)			
	OR	95% CI	P value		OR	95% CI	P value		OR	95% CI	P value
Subdural	Ref	–	–	Frontal	Ref	–	–	1 d	Ref	–	–
Subgaleal	0.95	0.31-2.87	.92	Parietal	1.27	0.62-2.60	.51	2 d	1.46	0.65-3.27	.36
Age											
Each year increase	1.01	0.98-1.03	.42		1.00	0.98-1.03	.85		1.00	0.98-1.03	.83
Gender											
Female	Ref	–	–		Ref	–	–		Ref	–	–
Male	2.85	1.23-6.60	**.01**		2.56	1.10-5.95	.03		2.49	1.06-5.84	**.04**
Preoperative GCS											
3-12	Ref	–	–		Ref	–	–		Ref	–	–
13-15	0.42	0.22-0.81	**< .01**		0.41	0.21-0.80	< .01		0.42	0.21-0.83	**.01**
Antiplatelet medication	0.63	0.27-1.43	.27		0.64	0.26-1.55	.32		0.66	0.28-1.54	.34
Warfarin	1.35	0.64-2.81	.43		1.51	0.71-3.23	.28		1.50	0.71-3.21	.29
								Preoperative maximal thickness			
								Each mm increase	1.02	0.99-1.05	.16
								Postoperative bed rest			
								No specific instructions	Ref	–	–
								1-12 h	0.31	0.04-2.54	.28
								12-24 h	1.46	0.69-3.09	.33
								24-48 h	0.96	0.34-2.70	.94
								48 + h	2.16	0.21-22.7	.52

OR, Odd's Ratio; CI, Confidence Interval; Ref, Reference. Bold values are statistical significance in P value.

**TABLE 4.** Multiple Logistic Regression Analysis With Adjusted Odds Ratios for Unfavorable Outcome (mRS 4-6) at Discharge in Patients According to Drain Location, Position, and Duration With Complete Data for Analysis

Location of drain (n = 562)				Position of drain (n = 508)				Drain duration (n = 530)			
	OR	95% CI	*P* value		OR	95% CI	*P* value		OR	95% CI	*P* value
Subdural	Ref	–	–	Frontal	Ref	–	–	1 d	Ref	–	–
Subgaleal	1.11	0.45-2.74	.83	Parietal	0.88	0.51-1.55	.67	2 d	1.66	0.93-2.94	.09
Age											
Each year increase	1.07	1.05-1.10	**< .01**		1.09	1.06-1.12	< .01		1.07	1.04-1.10	**< .01**
Gender											
Female	Ref	–	–		Ref	–	–		Ref	–	–
Male	0.71	0.44-1.16	.17		0.69	0.41-1.17	.17		0.71	0.43-1.17	.18
Admission mRS											
mRS 0-3	Ref	–	–		Ref	–	–		Ref	–	–
mRS 4-5	3.77	2.24-6.34	**< .01**		3.71	2.13-6.46	< .01		0.41	0.24-0.69	**< .01**
Pre-operative GCS									0.92	0.53-1.61	.78
3-12	Ref	–	–		Ref	–	–		0.71	0.37-1.35	.29
13-15	0.42	0.25-0.69	**< .01**		0.46	0.27-0.79	< .01				
Antiplatelet medication	1.11	0.65-1.87	.71		1.07	0.60-1.90	.82		Ref	–	–
Warfarin	0.80	0.44-1.48	.48		0.85	0.45-1.62	.63		3.80	2.24-6.46	**< .01**
Bed rest											
No specific instructions	Ref	–	–		Ref	–	–		Ref	–	–
1-12 h	1.36	0.46-3.97	.58		1.29	0.36-4.61	.69		1.51	0.51-4.46	.45
12-24 h	1.58	0.92-2.72	.10		1.63	0.90-2.95	.11		1.91	1.07-3.40	**.03**
24-48 h	1.87	0.94-3.70	.07		2.13	0.99-4.59	.05		1.88	0.92-3.84	.08
48 + h	8.30	1.00-688	**.05**		8.94	1.05-76.2	.05	*	–	–	–

OR, Odd's Ratio; CI, Confidence Interval; Ref, Reference. Bold values are statistical significance in *P* value.

**TABLE 5.** Postoperative Complications in Patients With Differing Drain Location and Position

	Drain location			Drain position		
Complication location	Subdural	Subgaleal	*P*-value	Frontal	Parietal	*P*-value
Surgical site infection	3 (0.6)	0 (0)	.62	1 (0.6)	2 (0.6)	.96
Seizures	8 (1.5)	2 (4.6)	.14	2 (1.1)	7 (2.0)	.43
New deficit	17 (3.2)	0 (0)	.23	9 (4.9)	6 (1.7)	**.04**
Respiratory infection	43 (8.1)	3 (6.8)	.77	13 (7.1)	28 (8.1)	.67
New arrhythmia	8 (1.5)	0 (0)	.41	2 (1.1)	6 (1.7)	.56
Venous thromboembolic event	2 (0.4)	0 (0)	.68	1 (0.6)	1 (0.3)	.65
Myocardial infarction	2 (0.4)	1 (2.3)	.09	0 (0)	2 (0.6)	.30
Cerebrovascular event	7 (1.3)	0 (0)	.44	4 (2.2)	3 (0.9)	.21
Death	9 (1.7)	0 (0)	.39	2 (1.1)	6 (1.7)	.56

Bold values are statistical significance in *P*-value.

**TABLE 6.** Postoperative Complications in Patients With Differing Drain Duration

	1 d (n = 157)	2 d (n = 392)	*P*-value
Surgical site infection	3 (1.9)	0 (0)	< .01
Seizures	2 (1.3)	7 (1.8)	.67
New deficit	3 (1.9)	13 (3.3)	.38
Respiratory infection	14 (8.9)	30 (7.7)	.62
New arrhythmia	0 (0)	7 (1.8)	.09
Venous thromboembolic event	0 (0)	2 (0.5)	.37
Myocardial infarction	0 (0)	3 (0.8)	.28
Cerebrovascular event	2 (1.3)	5 (1.3)	1.00
Death	5 (3.2)	4 (1.0)	.07

### Drain Duration

Only 4.9% of patients had drain insertion for at least 3 d, so were excluded from the subsequent analysis (Table 1). Analysis of patients with drain insertion for either 1 or 2 d demonstrated that recurrence rates were comparable between the 2 groups, 6.4% and 8.4%, respectively (*P* = .44, Table 2). There was no significant difference in mRS scores between the 2 groups (*P* = .56, Table 2), although on multivariate analysis there appeared to be a non-significant trend towards worse functional outcome with 2 d of drainage (OR 1.66, *P* = .09, Table 4).

## DISCUSSION

The location, position, and duration of drain insertion following burr-hole drainage of a chronic subdural hematoma do not affect symptomatic recurrence requiring repeat surgery, or functional outcome. This subgroup analysis comprises the largest study performed on drain position and duration in CSDH. The symptomatic recurrence rate we observed at 60 d may be underestimated if there were even later recurrences; however, previously published data suggest that recurrence is most likely within this time period.^6^ The median time to recurrence in the present study agrees with that reported previously.^7^

Where variation in practice does not impact on patient outcomes, there may be opportunities to simplify care pathways, reducing costs, and enhancing patient experiences. There are many operative and perioperative variables in the management of a patient with CSDH. In our previous multicenter cohort study, we demonstrated that drain insertion was an important predictor of outcomes. Other common variations in practice such as duration of postoperative bed rest and prescription of high-flow oxygen were not.^4^

### Drain Location

In their randomized controlled trial of drain use, Santarius and colleagues^1^ specified drain placement in the subdural space through either burr hole, kept in a dependent position, and removed at 48 h. They demonstrated significantly lower recurrence rates and improved 6-mo outcomes when compared to no drain use.^1^ Follow-up data have also demonstrated better long-term survival in the drainage group.^8^ However, the authors did not indicate the length of drain to be inserted intracranially, and postoperative imaging was not collected to confirm the position of the drain. Similarly, we did not confirm the drain position radiologically in our cohort and this analysis was dependent on details recorded in the operative notes.

Gazzeri et al^9^ and Zumofen et al^10^ reported retrospective medium-sized case series of extracalvarial (subperiosteal/subgaleal) drain insertion following BHC. Neither study had a subdural drain group for comparison, but the authors both reported recurrence rates comparable with the published literature. In a prospective, non-randomized study, Chih et al^11^ demonstrated no significant difference in recurrence rate and functional outcome. A small prospective randomized study purported no difference in recurrence rates but a better functional outcome in the subperiosteal drain group; this may have been confounded by the superior preoperative average mRS score in the subperiosteal group.^12^ By contrast, a retrospective single-center comparative study reported a higher re-operation rate for symptomatic recurrence in the subgaleal drain group compared to the subdural drain group, but there was a non- significant tendency to less-serious complications and lower 1 yr mortality in the subgaleal drain group.^13^ Similarly, Chih et al^11^ observed a non-significant increase in complications in the subdural drain group.

Perhaps the precise location of drain insertion in relation to the burr hole is not important. Without a standard definition of ‘subdural’ and ‘subgaleal’ drain position, it is possible that the drain positions may not differ that much; some drains described as being positioned in the subdural space may, in fact, be inserted a little distance beyond the burr hole, similar to drains positioned over the burr hole. Proponents of subgaleal drains argue that by avoiding instrumenting the subdural space, there should be a reduced risk of intracranial hemorrhage, brain injury, empyema, and epilepsy. We observed no significant difference in seizures between subdural and subgaleal drain groups. However, our results should be interpreted with caution given the small numbers of subgaleal drains used during the study period, and likely selection bias, as well as lack of prespecified data collection on intracranial hemorrhage and empyema. An adequately powered randomized controlled trial of subdural vs subgaleal drains is needed, one of which is underway in Switzerland.^14^

### Drain Position

Previous studies have also examined whether there is an advantage to frontal or parietal drain placement. Two small retrospective studies of patients undergoing BHC reported lower recurrence rate when the drain tip is placed in the frontal position within the subdural space.^15,16^ Our results suggest that it does not matter through which burr hole a drain is placed in terms of recurrence and functional outcome, although we observed a higher risk of new neurological deficit when the drain was placed via the frontal burr hole. It is difficult to ascertain the clinical significance of this finding because of the small number of events reported and the reporting of neurological deficit was not part of our primary outcome, therefore defined broadly and may be subjected to reporting bias. We are also unable to comment upon drain direction and ultimate tip position. Several small retrospective studies demonstrated no impact of the position of the intracranial subdural drain on recurrence rates.^17-19^

### Drain Duration

We observed no apparent advantage to drainage for more than 24 h and prompt drain removal may facilitate early mobilization, which, in turn, could reduce morbidity and improve functional outcomes. Previous studies examining the impact of duration of postoperative drainage on CSDH recurrence and functional outcomes are contradictory. Kale et al^20^ retrospectively compared recurrence rates after BHC in those drained for 2 to 4 d vs 5 to 7 d. They reported a significantly lower recurrence rate in the 5 to 7 d group (3.3% vs 15.6%), although the difference in actual reoperation rate between groups was much less stark (5% vs 2%). Yu et al^21^ reported significantly different recurrence rates in their retrospective series of 97 patients undergoing BHC (16.3% for patients drained <3 d vs 1.3% in those drained ≥3d), although presenting GCS scores were not recorded and may have differed between 2 groups. A randomized controlled trial comparing 48 h vs 96 h of postoperative drainage after twist-drill craniostomy (TDC) demonstrated no difference in recurrence rates, but increased mortality and complications and in the 96 h group.^22^ Sindou et al^23^ also performed a small randomized study comparing the same drainage durations in TDC and reported similar results. Jeong Si et al^16^ found no difference in recurrence rate when comparing <2 d and ≥2 d in BHC. Given the size and design of these studies, it is difficult to ignore the findings of the high-quality drain study from Santarius and colleagues^1^ that utilized a 48 h drainage period.^8^ The question of drain duration merits further study.

## CONCLUSION

Recurrence rate and functional outcome after drainage of primary CSDH by BHC does not appear to be affected by drain location, position, or duration of drainage. Choice of drain position and duration will be guided by surgeon preference and intraoperative findings, but variation in practice may not influence outcomes. Well-designed and adequately powered randomized controlled trials will help interrogate questions of drain position, location, and duration further.

### Disclosures

Dr Hutchinson is supported by a Research Professorship from the National Institute for Health Research (NIHR), the NIHR Cambridge Biomedical Research Centre, a European Union Seventh Framework Program grant (CENTER-TBI; grant no. 602 150), and the Royal College of Surgeons of England. Dr Kolias is supported by a Clinical Lectureship, School of Clinical Medicine, and University of Cambridge. The authors have no personal, financial, or institutional interest in any of the drugs, materials, or devices described in this article.

## Notes

This work was presented orally at the Autumn Society of British Neurological Surgeons’  Meeting, in Liverpool, United Kingdom, on September 21, 2017. The abstract only was published in the Proceedings of the 2017 Autumn Meeting of the Society of British Neurological Surgeons (British Journal of Neurosurgery).

## COMMENTS

The authors have evaluated the formidable question as to whether drain location, position, and duration impact outcomes in the management of CSDH population. They have conducted a prospective multicenter trial with a rigorous methodology and applied statistical analysis. The results add to our understanding of the impact of the use of drains, an important and frequently encountered clinical management issue.

**Odette Harris**

*Stanford, California*

Chronic subdural hematoma (CSDH) is an increasingly common disorder in neurosurgical practice. The present article is part of a multicenter, prospective cohort study of 577 CSDH patient treated in the UK. Subgaleal drains were uncommonly used and the study is thus underpowered to answer whether drain location (subgaleal or subdural) is a factor influencing recurrence and/or complication rates. Drain duration, 1 or 2 days, did not influence recurrence rates. This is an important finding, suggesting that earlier drain removal could be considered allowing faster mobilization of the patient. This well-written study demonstrates the benefits of nation-wide, multicenter surveys of the treatment of common neurosurgical diseases. The provided results are relevant for the neurosurgeon involved in the care of CSDH patients.

**Niklas Marklund**

*Lund, Sweden*
